# Increased reproductive outcomes after optimized sperm preparation

**DOI:** 10.3389/fcell.2025.1596421

**Published:** 2025-05-13

**Authors:** Matías D. Gómez-Elías, Guillermina M. Luque, Natalia Oscoz-Susino, Analía G. Novero, Olinda Briski, Inés Kásparas, Tomás J. Steeman, Cintia Stival, Mariano Lavolpe, Vanina Julianelli, Marisa Geller, Martín Attie, Rita Vassena, Darío Krapf, Mariano G. Buffone

**Affiliations:** ^1^ Fecundis Lab, Barcelona, Spain; ^2^ Instituto de Biología y Medicina Experimental (IBYME-CONICET), Buenos Aires, Argentina; ^3^ Instituto de Biología Molecular y Celular de Rosario (IBR, CONICET-UNR), Rosario, Argentina; ^4^ In Vitro Buenos Aires, Buenos Aires, Argentina

**Keywords:** assisted reproductive technology, capacitation, *in vitro* fertilization, implantation, live birth

## Abstract

A key factor to the success of *in vitro* fertilization (IVF) is the preparation of human sperm, a critical step that directly impacts the efficacy of the procedure. This proof-of-concept study evaluated the effect of HyperSperm, a novel sperm preparation technique designed to enhance sperm function, on fertilization, embryo development, and pregnancy outcomes in both a mouse model and a first-in-human trial following IVF. In mice, HyperSperm treatment significantly increased hyperactivated motility (p < 0.05), leading to improved fertilization and blastocyst development (p < 0.05), as well as higher implantation rates (p < 0.05) and larger litter sizes (p < 0.05). Offspring displayed normal growth and fertility. In human sperm samples from normozoospermic men, HyperSperm exhibited a high safety profile, with motility, acrosome reaction, viability, and DNA fragmentation comparable to controls. A first-in-human, prospective, single-center, split-oocyte study in 10 couples undergoing IVF with donated oocytes demonstrated similar fertilization rates between HyperSperm and control groups (p = 0.425), but significantly higher usable blastocyst rates in the HyperSperm arm (43.8% vs. 67.9%, p = 0.0122). Morphokinetic parameters were comparable between groups. These results suggest that HyperSperm enhances sperm hyperactivation, a hallmark of capacitation, leading to improved embryo development in both mice and humans. This technique represents a promising approach to optimizing sperm preparation in assisted reproduction, warranting further clinical investigation.

## Introduction

Human Assisted Reproductive Technologies (ART) have revolutionized the treatment of infertility, offering hope to millions of couples worldwide. ART techniques have evolved over the decades, leading to improved outcomes and expanding the scope of reproductive medicine. Despite these advancements, the success rates of ART procedures vary widely, due to factors such as maternal age, ovarian reserve, and embryo quality influencing outcomes (Banker et al., 2021; Chambers et al., 2021).

Current sperm preparation methods focus mostly on sperm selection based on motility characteristics, due to the association between appropriate sperm shape and progressive linear motility with fertility (Henkel and Schill, 2003). These methods have taken advantage of the abundance of sperm cells in an ejaculate, and have mostly focused on reproducing, by selection, the characteristics of a normospermic sample as defined by the WHO (World Health Organization, 2021). Unfortunately, morphology and swimming patterns, while important from a diagnostic point of view, are inaccurate proxies for the appropriate capacitation of sperm. Thus, selection based on these characteristics may not necessarily result in the most functional sperm sample, with potential repercussions on treatment effectiveness and efficiency (Bibi et al., 2023; Oseguera-López et al., 2019).

A key factor to the success of IVF is the preparation of human sperm, a critical step that directly impacts the efficacy of the procedure (Balli et al., 2022). Several methods have been developed to optimize sperm preparation for IVF, aiming to select high-quality sperm with optimal motility and morphology while minimizing contaminants that may impair fertilization. Traditional methods typically focus on the isolation of motile and morphologically normal sperm while removing the seminal plasma (Jeyendran et al., 2019; Vaughan et al., 2019; Pinto et al., 2020). Moreover, advancements in sperm selection technologies, such as microfluidic devices, have emerged as promising tools for isolating high-quality sperm (Meseguer et al., 2024).

We hypothesized that standard sperm preparation does not fully reproduce the events that take place in the female reproductive tract. Ejaculated sperm must undergo a series of biochemical changes to attain fertilizing capacity (Austin, 1951; Chang, 1951). These modifications occur as the sperm travels through the uterus and oviduct, relying on signaling pathways involving ion channels and transporters such as CatSper (Ren et al., 2001), Hv1 (Lishko et al., 2010) or SLO3 (Navarro et al., 2007), many of which are uniquely expressed in sperm (Puga Molina et al., 2018) and respond to changes in pH and ion concentrations in the female tract (Beltrán et al., 2016; Wang et al., 2021). This dynamic environment is not replicated effectively *in vitro* during IVF, where sperm cells are incubated in a unique medium of defined composition.

The sperm contribution to post-fertilization events has been traditionally considered limited to providing paternal DNA, activating the oocyte through the PLCzeta protein (Saunders et al., 2002), and, depending on the species, contributing the proximal centriole (Avidor-Reiss et al., 2020). However, growing evidence suggests that the sperm role extends beyond these actions, as manipulation of sperm signaling pathways, metabolism, proteins or RNA content can influence embryo development before implantation (Liu et al., 2012; Tateno et al., 2013; Navarrete et al., 2019; Tarozzi et al., 2021; Vallet-Buisan et al., 2023).

In an effort to recapitulate *in vitro* the *in vivo* capacitation process, we developed a procedure for preparing sperm for IVF, which involves sequential steps of incubation in different media to promote the activation of signaling pathways crucial for sperm capacitation. We tested whether this procedure, named HyperSperm, is associated with better reproductive outcomes both in a mouse model of IVF and in the human reproductive clinic.

## Results

### HyperSperm enhances sperm hyperactivation and improves reproductive outcomes in a mouse model of IVF

**FIGURE 1:**
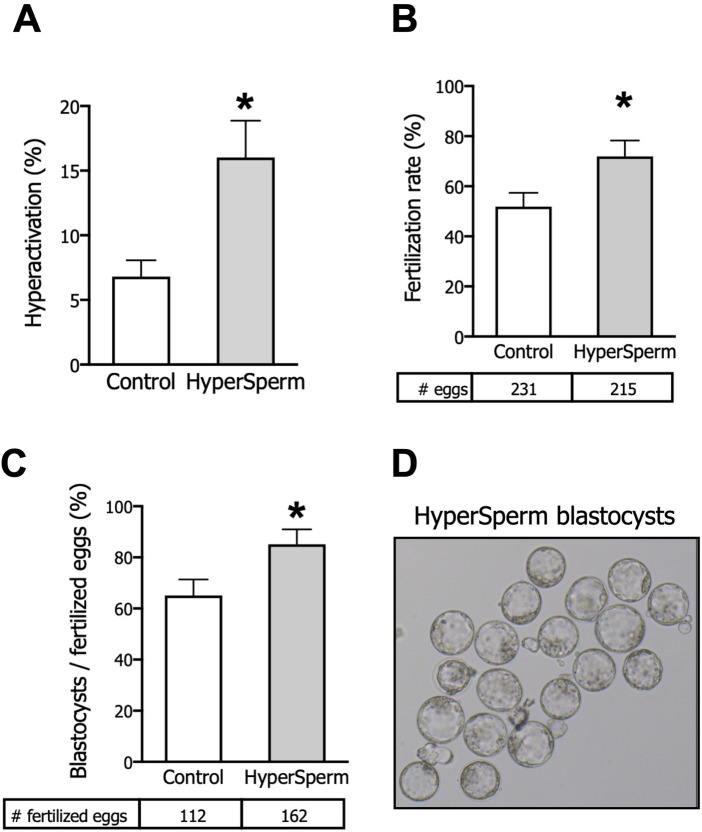
HyperSperm increases fertilization and embryo development rates. **(A)** Hyperactivation, measured by CASA, n = 5. **(B)** Fertilization rates (2-cell embryos/eggs), n = 10. **(C)** Embryo development rates (blastocysts/fertilized eggs), n = 10. **(D)** Representative image of HyperSperm-derived blastocysts. Paired t-test was performed, *p < 0.05 represents statistical significance vs. control.

In a mouse model, HyperSperm did not affect total sperm motility compared to the Control (Supplementary Table S1), but did produce a significant increase in the percentage of hyperactivated cells (Figure 1A) as well as in the kinematic sperm parameter VCL (Supplementary Table S1).

We next tested whether HyperSperm led to higher fertilization rates, and found that HyperSperm-treated sperm gave rise to a higher number of 2-cell stage embryos when incubated with COCs from F1 females (Figure 1B). More importantly, a higher percentage of 2-cell embryos progressed to the blastocyst stage when sperm were treated with HyperSperm compared to Controls (Figure 1C). Representative images of HyperSperm-derived blastocysts are displayed in Figure 1D.

**FIGURE 2:**
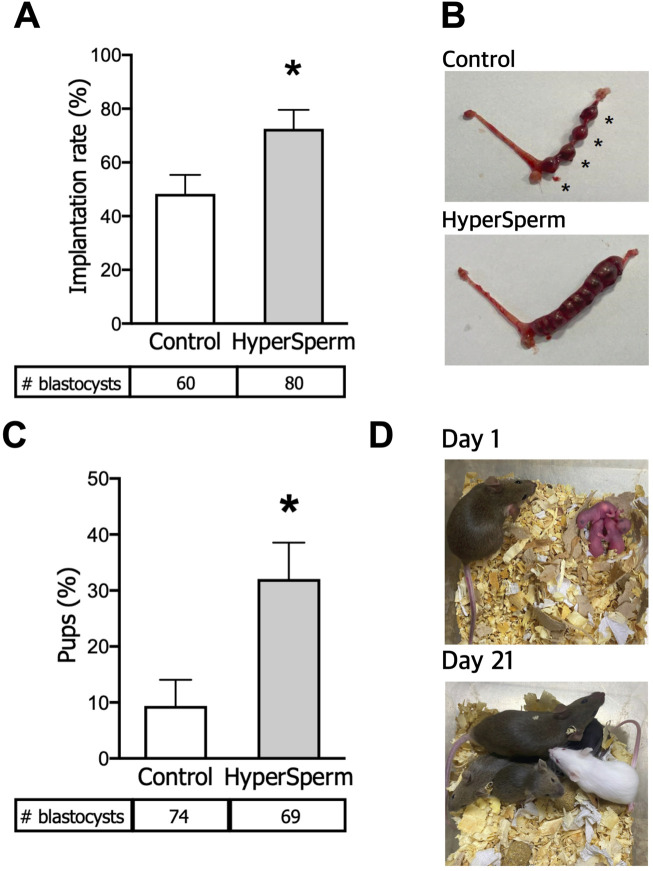
HyperSperm increases the efficiency of embryo implantation. **(A)** Implantation rates (implantation sites at day 7/blastocysts transferred), n = 6 for Control and n = 8 for HyperSperm. **(B)** Representative image of implantation sites (*) obtained with Control (upper panel) and HyperSperm (lower panel) treatments. **(C)** Litter size (% of pups born per blastocyst transferred), n = 8 for Control and n = 7 for HyperSperm. **(D)** Representative image sequence of HyperSperm pups on different days after birth. Data represents the mean ± SEM. Unpaired t-test was performed, *p < 0.05.

We next assessed the implantation and developmental potential of these blastocysts. Eight to ten blastocysts from each arm were non-surgically transferred to pseudo-pregnant females. The implantation status was recorded 7 days post embryo transfer. The number of implantation sites was higher when the embryos transferred were derived from HyperSperm compared to the Control (Figure 2A). Representative images of implantation sites are provided in Figure 2B.

To further evaluate pregnancy progression, several parameters were assessed from an equal number of non-surgically transferred blastocysts derived from Control or HyperSperm-treated sperm (8–10 in each arm). The number of live pups born was 0.9 ± 1.2 vs. 3.1 ± 1.7, respectively (n = 7-8 recipient females, p < 0.05, Mann-Whitney test); the observed increase in HyperSperm pups was reflected in an increased ratio of transferred embryos producing offspring (Figure 2C). Representative images of HyperSperm pups’ growth after birth are presented in Figure 2D.

### HyperSperm exhibits an excellent safety profile in pregnancy and beyond in mice

**FIGURE 3:**
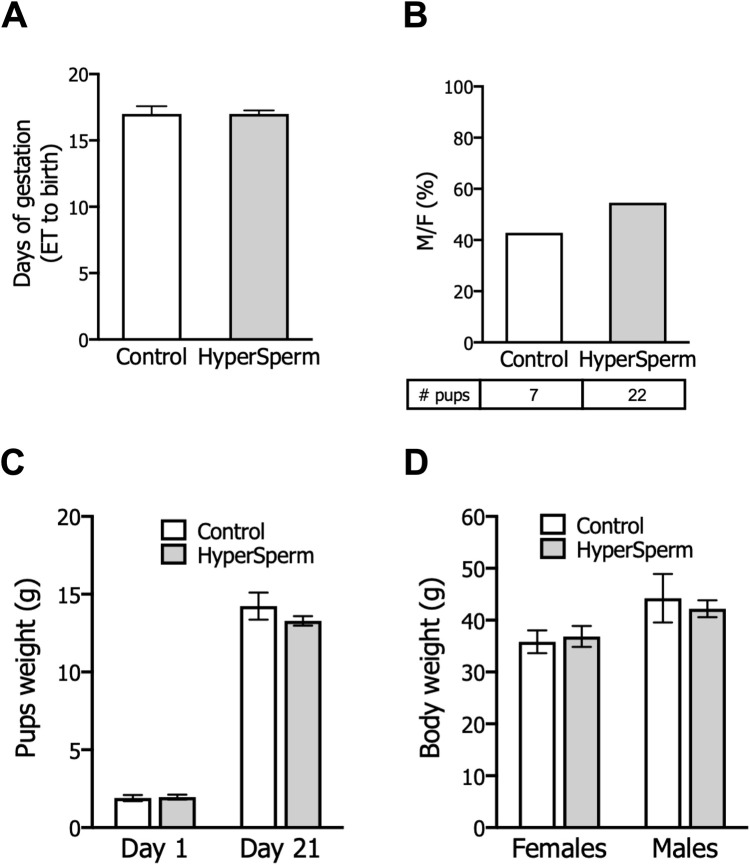
HyperSperm produces pups of normal weight. **(A)** Gestation period (days from embryo transfer to birth). Data represents the mean ± SEM, n = 3 litters for Control and n = 6 litters for HyperSperm. Unpaired t-test was performed. **(B)** Percentage of males born relative to the total offspring for each treatment. Data represents the mean. **(C)** Pup weight at birth (day 1) and at weaning (day 21). Data represent the mean ± SEM. **(D)** Body weight of females and males at 6 months. Data represent the mean ± SEM.

**FIGURE 4:**
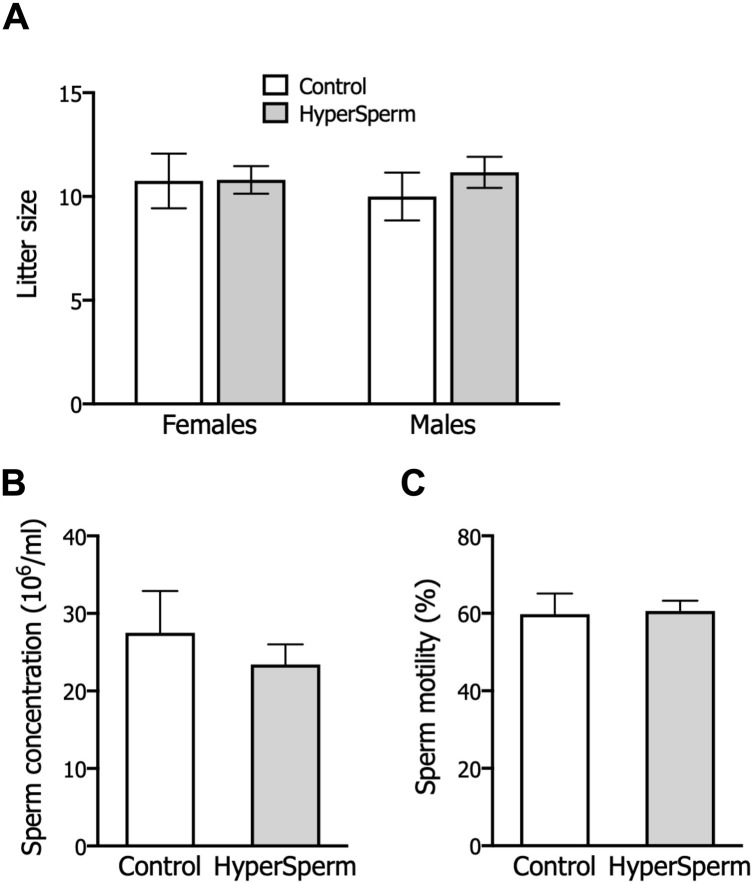
HyperSperm generates fertile progeny. **(A)** Litter size (number of pups/litter) obtained by mating Control or HyperSperm-derived females or males with hybrid F1 mice. **(B)** Sperm concentration of male born as a result of treatment. **(C)** Sperm motility (%) of male born as a result of treatment. In all cases, data represents the mean ± SEM of at least 3 matings, and Student’s t-test was performed.

In mice, pregnancy duration was unchanged between females that received embryos from either group (Figure 3A), and the proportion of females and males in litters generated with HyperSperm was similar to those in the Control group (Figure 3B). Additionally, the body weight of HyperSperm-derived pups at birth (day 1), weaning (day 21), and sacrifice (6 months) were comparable to those resulting from Control (Figures 3C,D). Furthermore, HyperSperm-derived mice were fertile, as mating of HyperSperm-derived males or females with F1 mice resulted in normal-sized litters (Figure 4A). Additionally, sperm from HyperSperm-derived mice were subjected to *in vitro* analysis to detect potential differences that could have been overlooked under *in vivo* conditions. Sperm analysis showed no differences in cauda epididymal sperm count and motility (Figures 4B,C) between Control- and HyperSperm-derived mice.

### HyperSperm results in a higher percentage of hyperactivated human sperm without negative effects

**FIGURE 5:**
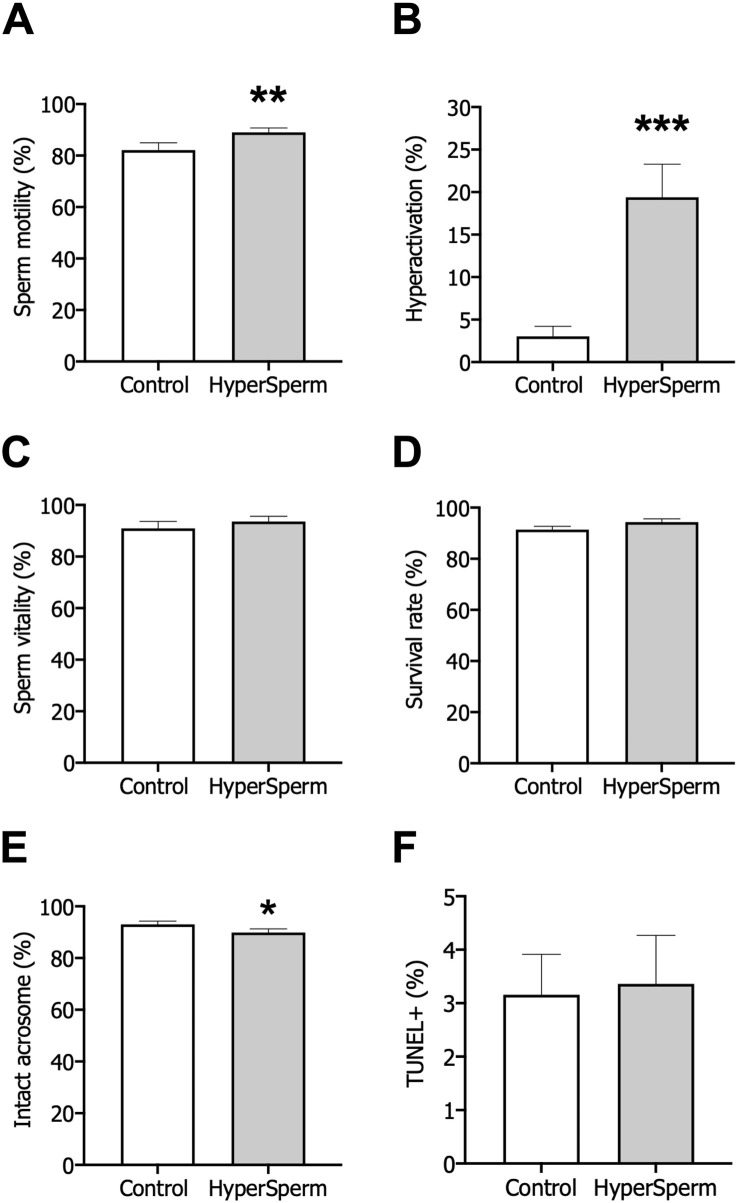
Assessment of safety after treatment with standard conditions (Control) or HyperSperm. **(A)** Total motility, measured by CASA, n = 12. **(B)** Hyperactivation, measured by CASA, n = 12. **(C)** Percentage of viable cells, using eosin-Y staining, n = 9. **(D)** Survival measured as sperm vitality after overnight incubation, n = 5. **(E)** Acrosomal integrity, using FITC-PSA staining, n = 14. **(F)** DNA fragmentation, using the TUNEL assay, n = 17. Wilcoxon matched-pairs signed rank test was performed, *p < 0.05; **p < 0.01; ***p < 0.005.

Based on the results obtained in the mouse model, we decided to proceed with *in vitro* studies on human sperm. In human samples, HyperSperm did not affect total sperm motility compared to control (Figure 5A), and confirmed the increase in the percentage of hyperactivated cells (Figure 5B) and several kinematic sperm parameters such as VCL, VAP, ALH and BCF (Supplementary Table S2).

Subsequently, we assessed the safety of HyperSperm on human sperm. First, the percentage of live cells was unaffected by HyperSperm treatment compared to the Control (Figure 5C). Further, the percentage of cells that remained viable after an overnight incubation remained unchanged between the two arms (Figure 5D). Then, we assessed acrosome loss and found that the percentage of intact acrosomes was clinically comparable in the two groups (Figure 5E). Finally, DNA fragmentation was not induced by HyperSperm when compared to Control (Figure 5F). Overall, HyperSperm significantly increased the proportion of hyperactivated cells, a parameter associated with capacitation, without impairing the functionality and quality of human sperm.

### HyperSperm improves reproductive outcomes in patients

Based on our preclinical studies in mice and the safety studies in human sperm, we explored whether HyperSperm improves IVF outcomes in a prospective first-in-human study with split design in 10 couples. The main characteristics of the patients and donors enrolled in this study are presented in Table 1. From 70 assigned COCs in each arm, there were 3 and 7 immature oocytes on D1 in the HyperSperm and Control group, respectively.

**TABLE 1:** Age and seminal parameters from the participants of the pilot sibling oocyte study (n = 10).

Participants’ age	
Male partner age	40.9 ± 3.8 (33–46)
Recipient age	41.8 ± 2.7 (36–44)
Donor age	26.6 ± 2.2 (23–30)
Semen parameters	
Semen Volume (mL)	3.0 ± 1.7 (1.0–6.8)
Sperm concentration (x10^6^/ml)	112 ± 95 (14–320)
Morphology (%)	9.0 ± 5.1 (5.0–21.0)
Total motility (%)	66 ± 11 (50–78)
Progressive motility (%)	59 ± 15 (35–78)

Values are expressed as mean ± SD (range).

**FIGURE 6:**
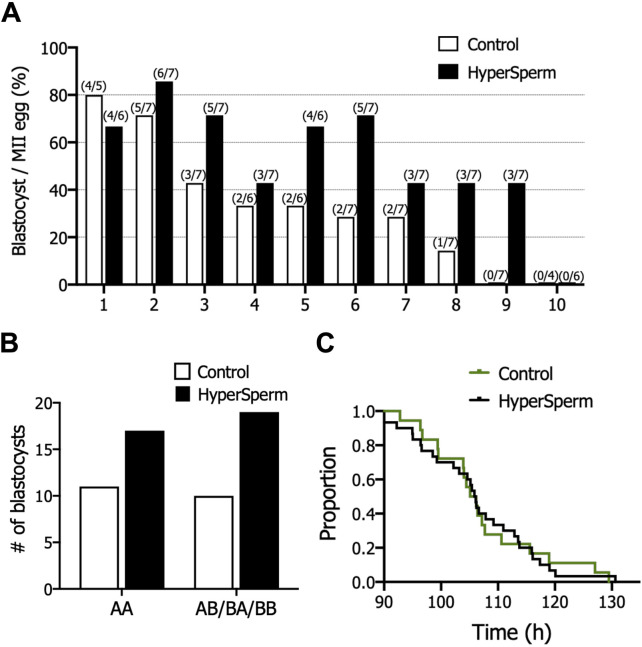
HyperSperm increased blastocyst production in humans. **(A)** Effect of HyperSperm on individual outcomes. Bars indicate the percentage of blastocysts per mature egg for each patient, ordered from the highest (1) to the lowest (10) value in the Control arm. **(B)** Effect of HyperSperm on embryo quality. Bars indicate the total number of blastocysts obtained in each group according to the Gardner grading system. AA: top quality embryos; AB/BB/BA: high quality embryos. Fisher’s exact test was performed, p-value = 0.787. **(C)** Effect of HyperSperm on embryo morphokinetics. Median time point at which embryos reached blastocyst stage in the Control and HyperSperm groups. Median t_B_: 106.2 vs. 106.0 h (Control vs. HyperSperm), n = 18 Control and n = 32 HyperSperm-derived blastocysts, p > 0.05.

The fertilization rate was comparable between Control and HyperSperm (76.1% vs. 79.1%, p = 0.425) (Table 2). However, the usable blastocyst rate, i.e., the percentage of blastocysts per fertilized egg that reached a Gardner score of 3BB and above, was significantly higher in the HyperSperm group (43.8% vs. 67.9%; p = 0.0122) (Table 2). The improvement observed in IVF parameters after using HyperSperm in each couple is shown in Figure 6A. HyperSperm increased blastocyst yield in 8/10 cases (Figure 6A), with an average of 1.5 more blastocysts available for each patient (2.1 vs. 3.6 blastocysts per patient).

**TABLE 2:** Embryo data following IVF with sperm treated with standard procedures (Control) or HyperSperm in 10 couples.

	Control	HyperSperm	*p-*value^a^
Number of COCs	70	70	
Number of oocytes after excluding immature oocytes on D1	63	67	
Fertilization rate (2PN embryos/mature eggs)	48/63 (76.1%)	53/67 (79.1%)	0.425
Usable blastocyst rate (usable blastocysts/2PN embryos)	21/48 (43.8%)	36/53 (67.9%)	0.0122

^a^ Statistical analysis using Fisher’s exact test.

Values are presented as number (%) from the total of n = 10 cases.

COCs, cumulus-oocyte complexes.

The proportion of top quality (AA, grade 3 or above) and good quality (AB/BA/BB grade 3 or above) blastocysts was similar between groups (Figure 6B). We further evaluated embryo development with time-lapse monitoring. Morphokinetic development of HyperSperm blastocysts was not different from Control for all parameters evaluated (Supplementary Table S3), including time to blastocyst (tB, Figure 6C).

Although this study was not designed to explore clinical outcomes beyond embryo development, some blastocysts derived from HyperSperm were transferred based on the embryologists’ decision. This resulted in three normal pregnancies and the live birth of healthy boys at the time of writing (2 by cesarean delivery and 1 by vaginal delivery, and weighing at birth: 3020, 3800 and 3525 g, respectively).

## Discussion

The incubation of sperm under capacitating conditions was the cornerstone of successful IVF in rodents (Toyoda and Chang, 1974) and a tremendous step forward for the success of IVF in humans (Steptoe and Edwards, 1978). However, these conditions are still far from optimal. For instance, IVF typically requires thousands of sperm to achieve fertilization rates of >70% while *in vivo* fertilization in mice is achieved at a sperm-to-egg ratio of ∼1 in the ampulla (Stewart-Savage and Bavister, 1988; La Spina et al., 2016) or with ∼40 sperm in humans (Williams et al., 1993). Additionally, proteins such as FER (Alvau et al., 2016), ZP3R/sp56 (Cheng et al., 1994), zonadhesin (Tardif et al., 2010), believed to be essential for fertilization based on *in vitro* experiments, do not exhibit significant phenotypes *in vivo* when knocked out (Okabe, 2018).

Several factors contribute to the poor efficiency observed *in vitro* compared to the natural processes in the female reproductive tract: intense selection at the uterotubal junction (Holt and Fazeli, 2010); exposure to oviduct and uterus secretions, which contain a variety of compounds not present in the media used for capacitation; and changing conditions along the reproductive tract, with ions and proteins present in gradients of concentrations rather than in fixed amounts (Killian, 2011).

Here, we show that the modulation of sperm capacitation, to the extent that it recapitulates *in vivo* events, can contribute to the preimplantation development of high-quality embryos and, ultimately, to the birth of a healthy offspring. We confirm this effect in two mammalian species, strengthening our findings.

Specifically, we observed a more robust developmental competence of embryos derived from HyperSperm-treated sperm, reaching the blastocyst stage in higher numbers without an apparent alteration in morphokinetics compared to controls.

In mice, 89% of the embryos produced by HyperSperm that reached the embryonic genome activation (EGA) stage were able to execute it appropriately and proceed to the blastocyst stage, a significant increase from the 61% of the control. Likewise, when analyzing the developmental dynamics in human embryos, we found that they seem to better withstand the maternal to embryonic transition which reaches its peak at D3 of development (Vassena et al., 2011). Although the mechanistic explanation for this observation is still under investigation, recent evidence points to the possibility of non-genetic sperm mediated effects on preimplantation embryo development (Guo et al., 2017; Tomar et al., 2024). It is conceivable that an appropriate capacitation could contribute to the mobilization of signaling molecules from the sperm such as cAMP, Ca^2+^ or K^+^. Intracellular Ca^2+^ levels play a pivotal role in sperm capacitation. Our methodology is based on the modulation of intracellular Ca^2+^ levels of sperm cells, to optimize sperm capacitation. Ca^2+^ acts as a key second messenger in regulating various physiological changes during capacitation, including hyperactivation of motility (Sánchez-Cárdenas et al., 2018), membrane fluidity alterations, and the activation of signaling cascades essential for the acrosome reaction (Buffone et al., 2014). In this regard, elevated intracellular Ca^2+^ concentrations modulate the activity of Ca^2+^-sensitive enzymes (Navarrete et al., 2015) and ion channels, promoting changes in membrane potential (Novero et al., 2024) that are critical for sperm to navigate the female reproductive tract and interact with the oocyte. As such, tight regulation of intracellular Ca^2+^ is essential for ensuring successful fertilization which in turn could provide a more functional environment to drive preimplantation development past the maternal contribution.

In mice, these embryos are also significantly more likely to implant and to develop to term. The design of our first-in-human study does not allow for a similar assessment in patients, although the 3 pregnancies that were established all resulted in live birth. It remains to be seen whether an increase in implantation ability is maintained across species.

An important aspect to consider when introducing a new technique in IVF is its safety. While innovations are increasingly available to clinicians and patients, the safety of many of them cannot be established even after their introduction in clinical practice (Lundin et al., 2023). Here we show an extensive safety assessment of HyperSperm in an animal model of IVF, with data reaching the second generation after the application of the technique. We further show that the technique is safe by all parameters analyzed, both at the cellular and at the molecular level, in human semen, while more extensive data on embryo development and pregnancy are being collected.

We recognize some shortcomings in this work. Chiefly, the small number of patients included in the human IVF study, whose results will need confirmation in a larger dataset. Nevertheless, the sibling oocytes, split design, and the consistency of the outcome across the vast majority of patients provides encouragement that the differences observed in embryo development are indeed due to HyperSperm.

Access to fertility care is a key issue globally. One of the most frustrating experiences for clinicians is the knowledge that treatments may work with time, but that patients often lack the psychological and financial resilience to withstand multiple cycles of stimulation and embryo transfers. At the clinic level this translates in a much smaller reach of the care provided, compared to the patient population needing help to conceive. It is our hope that HyperSperm may contribute to facilitating access to care by increasing the number of embryos available for transfer. This would result in a higher proportion of patients able to complete their treatment as intended in the first cycle, with a shorter time to live birth and lower dropout rates.

## Materials and methods

### Reagents

All reagents and chemicals were purchased from Sigma-Aldrich Chemicals (St Louis, MO, United States), unless otherwise specified.

### Mice

Hybrid F1 (BALB/c female x C57BL/6 male) mature (10–12 weeks-old) male and female mice were used. In all cases, mice were housed in groups of 4 or 5 in a temperature-controlled room (23°C) with lights on at 07:00 h and off at 19:00 h and had free access to tap water and laboratory chow. All experimental procedures were carried out according to institutional animal care guidelines and were reviewed and approved by the Ethical Committees of the Instituto de Biología y Medicina Experimental, Buenos Aires #24/2021. Experiments were performed strictly following the Guide for Care and Use of Laboratory Animals approved by the National Institutes of Health (NIH).

### Mouse sperm collection and incubation

The non-capacitating medium used in this study was a modified Toyoda–Yokoyama–Hosi (modified TYH) containing 119.3 mM NaCl, 4.7 mM KCl, 1.71 mM CaCl_2_·.2H_2_O, 1.2 mM KH_2_PO_4_, 1.2 mM MgSO_4_. 7H_2_O, 0.51 mM sodium pyruvate, 5.56 mM glucose, 20 mM HEPES and 10 μg/mL gentamicin (NC medium). For capacitating conditions, 15 mM NaHCO_3_ and 5 mg/mL bovine serum albumin (BSA) were added (CAP medium). In all cases, pH was adjusted to 7.4 with NaOH.

Animals were euthanized and cauda epididymal mouse sperm were collected. Both cauda epididymides were placed in 0.7 mL of NC medium for 15 min at 37°C (swim-out). In the Control group, sperm were capacitated for 90 min at 37°C in 200 µL CAP medium to reach a final concentration of 5–10 × 10^6^/mL. In the treated group, the protocol consisted of sequential incubation steps carried out through 3 proprietary media of defined composition, each protected by intellectual property and designed to modulate the levels of intracellular Ca^2+^ (HyperSperm).

### Mouse sperm motility analysis

After incubation in the appropriate medium, mouse sperm suspensions were loaded on a 46 μm deep chamber and placed on a microscope stage at 37°C. Sperm movements were examined using a Computer-Assisted Semen Analysis (CASA) system (Hamilton Thorne Ivos I, Beverly, MA, United States). Parameters used were as follows: 30 frames acquired at 60 Hz, and cell size of 30–170 μm^2^. At least 200 sperm were analyzed in each experiment. The following parameters were measured: mean path velocity (VAP), curvilinear velocity (VCL), straight-line velocity (VSL), linearity (LIN), amplitude of lateral head displacement (ALH), and straightness (STR). Sperms were considered hyperactivated when presenting VCL ≥271 μm/s, LIN <50%, and ALH ≥7 μm.

### Mouse egg collection and IVF assay

Ten to 12-week-old F1 female mice were superovulated using equine chorionic gonadotropin (5 IU, eCGPMSG; Syntex, Argentina) administered at 18:30 h, followed by human chorionic gonadotropin (5 IU, hCG; Syntex, Argentina) intraperitoneal injection 48 h later (18:30 h). Cumulus oocyte complexes (COCs) were collected from oviducts 14–15 h post-hCG administration and placed in TYH IVF medium (which contains 25 mM NaHCO_3_ and 4 mg/mL BSA, without HEPES addition). Fertilization droplets containing 10–30 COCs were inseminated with sperm incubated with either Control or HyperSperm treatment, at a final concentration of 0.5 × 10^6^/mL (at 11:00 h, day 0, D0). After 4 h, eggs were washed and placed in fresh media (15:00 h, D0). Fertilization was assessed by visualization of 2-cell embryos on the following day (24 h after insemination, 11:00 h, D1) under a stereoscopic microscope Nikon SMZ800 (Nikon, Japan).

### Mouse embryo culture and embryo transfer

Twenty-four hours post-insemination fertilized 2-cell embryos were transferred to droplets containing KSOM media (11:00 h, D1) and further incubated for 3 days (assessed at 10:00 h, D4). At this stage, the percentage of blastocyst formation was evaluated under the stereoscopic microscope. In some cases, 8 to 10 blastocysts were transferred (14:00 h, D4) to 2.5 days post coitum pseudo-pregnant F1 recipient females using the non-surgical uterine embryo transfer (NSET) device (Bin Ali et al., 2014). Pseudo-pregnant F1 recipient females were obtained by mating with vasectomized males 1 day after IVF (17:00 h, D1). Only females with a clear plug the following morning (on D2, 11:00 h) were chosen as embryo recipients. Embryo transfers were conducted in pairs, with Control and HyperSperm groups performed on the same days. Primed females were randomly assigned to either the Control or HyperSperm groups, and all embryo transfers were carried out in a blinded manner. The efficiency of embryo transfer was assessed by: 1) number of implantation sites at day 7 post embryo transfer; and 2) number of live pups born.

### Progeny studies

Body weight: Body weight of the pups born as the result of embryo transfer was recorded at weaning (day 21). Fertility test: Mature male and female mice born after embryo transfer were mated with hybrid F1 females or males, respectively. Litter size (number of pups/litter) was recorded. Sperm parameters: Sperm parameters from male mice derived from treatment were determined. To evaluate motility, sperm suspensions were placed on pre-warmed slides and analyzed subjectively under a light microscope (400×). We considered progressive motile sperm those cells that moved in a forward direction. We did not include in this category those sperm that vibrated or rotated in the same place. For sperm count, after swim-out, sperm suspensions were diluted in water to prevent sperm movement, and the number of sperm heads was recorded by a standard method using a Neubauer Chamber under a light microscope (400×).

### Ethical approval and consent

All procedures involving human semen samples and the participation of human subjects in the clinical first-in-human study were approved by the Ethics Committee of the IBYME-CONICET (Ref: CE 001/April 2019). All subjects were informed about the details of their participation and the study objectives, and given time to consider their participation. Signed consent was obtained from all participants before their inclusion in the study. Participant selection and inclusion were carried out by the ART clinic *In Vitro* Buenos Aires.

### Processing of human semen samples for *in vitro* assays

Semen samples of 17 normozoospermic men obtained by masturbation following abstinence of 2–7 days were directly placed into sterile containers and sent to the lab within 1 h. Samples were divided into two halves for Control (standard procedure) or HyperSperm processing. First, sperm were processed by density gradient centrifugation (PureSperm; Nidacon, Sweden). Sperm concentration was adjusted to 10–20 × 10^6^/mL. Control sperm were incubated in modified Human Tubal Fluid (modified HTF) medium (FUJIFILM Irvine Scientific, Santa Ana, CA, United States) containing 10% Serum Substitute Supplement (SSS, FUJIFILM Irvine Scientific) at 25°C for up to 4 h. In the experimental group, sperm were processed with HyperSperm. At the end of either Control or HyperSperm treatment, the following safety assays were conducted: sperm vitality, survival, DNA fragmentation and acrosomal integrity. All methods were performed according to the WHO Manual (World Health Organization, 2021).

### Assessment of sperm vitality and survival

Sperm vitality was assessed by dye exclusion using 0.5% Eosin Y. Sperm vitality was calculated as the number of sperm cells that did not incorporate the dye over the total number of cells in the field. For survival assessment, aliquots of sperm were incubated at room temperature overnight and vitality was assessed the following morning as described before.

### Assessment of DNA fragmentation

DNA fragmentation was evaluated by TUNEL assay (Roche Diagnostics GmbH, Mannheim, Germany) according to manufacturer instructions. Slides were analyzed in a fluorescence microscope (Primo Star iLED, ZEISS, Göttingen, Germany). The percentage of cells with fragmented DNA was determined by analyzing the number of sperm with green fluorescence (positive TUNEL) over the total number of sperm in each field. For each slide, 400 sperm cells were counted.

### Assessment of acrosomal integrity

The acrosomal integrity was evaluated by staining sperm cells with fluorescein isothiocyanate (FITC) labeled - *Pisum sativum* (PSA). Slides were analyzed in a fluorescence microscope (Primo Star iLED). The percentage of acrosome-intact sperm was calculated as the number of cells with a bright, uniform staining over the acrosome over the total of cells in each field. For each slide, 400 sperm cells were counted.

### CASA of human sperm

For motility assessment, aliquots of 9.7 µL of human sperm suspensions were placed on slides under 18 mm × 18 mm coverslips, obtaining a preparation depth of 30 µm depth chambers, and maintained at 37°C using a temperature-controlled stage. Sperm motility parameters were evaluated using the Sperm Class Analyzer® system (SCA v.6.2.0.1., Microptic SL, Barcelona, Spain), acquiring 60 frames per second. At least 5 microscopic fields and 300 sperm were analyzed. The kinematic parameters measured in the mouse were assessed. Sperm motility was measured and classified as follows: rapid progressive (VCL ≥35 μm/s; STR ≥80%), medium progressive (VCL ≥15 μm/s; STR ≥80%), *in situ* (VCL <15 μm/s; VAP ≥5 μm/s) and immotile (VAP <5 μm/s). Percentages of total (rapid progressive + medium progressive + *in situ*) and progressive (rapid + medium progressive) motility were recorded. Drifting was set at 25 μm/s. Sperm cells were considered hyperactivated (HA) when presenting VCL ≥150 μm/s, LIN <50% and ALH ≥3.5 µm.

### First-in-human study

This was a first-in-human sibling oocyte study including 10 couples attending *In vitro* Buenos Aires, between November 2021 and July 2022. The inclusion criteria were: women age 20–45 years, use of donated oocytes, men age 20–50 years, with the following sperm parameters after discontinuous density gradient: sperm count after swim-up ≥5 × 10^6^, motility ≥40%, normal morphology (Kruger criteria) ≥5%.

Exclusion criteria for women were: endometrioma or other uterine pathologies, any diagnosed sexually transmitted infection (STI), diabetes or other metabolic diseases, repeated pregnancy loss (>2 clinical pregnancies without live birth), abdominal surgery (diagnostic laparoscopy, hysteroscopy or surgical hysteroscopy were permitted), report of smoking, nicotine or marijuana use in the preceding 12 months, and abnormal vaginal bleeding without diagnosis at inclusion. Exclusion criteria for men were any diagnosed STI, and previous IVF failure.

### Ovarian stimulation and oocyte retrieval

Donors were stimulated with follitropin alpha (Folitime; GemaBiotech, Argentina or Gonal-F; Merck, Argentina), and/or corifollitropin alpha (Elonva; Organon, Argentina). When at least one follicle ≥14 mm of diameter was observed, pituitary suppression was initiated with a GnRH antagonist (0.25 mg of cetrorelix acetate, Cetrotide; Merck, Argentina; or 0.25 mg of ganirelix, Orgalutran®; Organon). Oocyte maturation was triggered with 0.1 mg of triptorelin acetate (Gonapeptyl Daily Decapeptyl; Ferring, Argentina), when at least 3 follicles of diameter ≥18 mm were detected. Ovum pick-up was carried out 36 h after trigger, under ultrasound-guided transvaginal follicular aspiration. Fourteen COCs were assigned to each participating couple, 7 in each arm. COCs were transferred to IVF medium (SAGE, CooperSurgical, Trumbull, CT, United States) and incubated at 37°C, 7% CO_2_ and 5% O_2_ for up to 4 h before insemination. Surplus COCs were denuded and MII oocytes were vitrified for future clinical procedures, unrelated to this trial.

### Sperm samples preparation

Semen samples of male patients were obtained in the clinic by masturbation into sterile containers and ejaculates were liquified for up to 1 h at room temperature prior to processing. Samples were divided into two halves for Control (standard procedure) and HyperSperm processing. After sperm selection, Control sperm were incubated in IVF medium (SAGE) at 25°C while the experimental group was processed with HyperSperm.

### *In vitro* fertilization

IVF was performed in 30 μL drops of IVF medium (SAGE), supplemented with 10% serum (SAGE) and under mineral oil (SAGE). COCs were inseminated with Control or HyperSperm-treated sperm, and incubated at 37°C, 7% CO_2_ and 5% O_2_. The number of fertilized eggs (i.e., with two pronuclei, 2PN) was recorded 18–20 h after insemination. Fertilization rate was calculated after excluding immature oocytes on D1 as the percentage of 2PN per mature egg.

### Embryo culture and time-lapse monitoring

Fertilized eggs were cultured in Esco Miri® TL (Esco Medical, Denmark) incubator for time-lapse monitoring for 5–6 days to blastocyst stage. Cells were incubated individually in Global Total medium (LifeGlobal, CooperSurgical) under mineral oil (SAGE) at 37°C, and under 7% CO_2_ and 5% O_2_ atmosphere. Embryo assessment was performed during the morning of day 5 and 6, and the number of high-quality blastocysts was recorded. Usable blastocysts were those with a score of 3BB or above according to the Gardner grading system (Gardner and Balaban, 2016). If Gardner’s score was lower than 3BB at day 5 (D5), embryos were incubated one additional day (D6). Blastocyst rate was calculated as the percentage of blastocysts per fertilized egg. Time-lapse monitoring allowed the determination of the time of each developmental event: tPNf (time of pronuclei fading); t2 (time to 2 cells); t3 (time to 3 cells); t4, (time to 4 cells); t5 (time to 5 cells); t8 (time to 8 cells); tM (time to morula); tB (time to blastocyst); cc2 (duration of the second cell cycle); cc3 (duration of the third cell cycle); s2 (time to complete synchronous division). Blastocysts were either vitrified or transferred according to physician and couple decisions. Embryo transfers were performed with one high-quality blastocyst each time, chosen by quality.

### Statistical analysis

Statistical analyses were performed using the GraphPad Prism 6 software (La Jolla, CA, United States). For mouse studies, at least 3 independent experiments were carried out using different mice. Parametric or non-parametric comparisons were used as dictated by data distribution. The specific statistical analysis employed is indicated in the relevant figure legend. In human studies, statistical differences between Control and HyperSperm groups were analyzed using Wilcoxon matched-pairs signed rank test for safety analysis, Fisher’s exact test for fertilization and embryo development, or a log-rank test (Mantel-Cox) for developmental times. Differences were considered significant when p < 0.05.

## Significant statement

HyperSperm enhances sperm hyperactivation, improving fertilization in mice and embryo development in both mice and humans. In a first-in-human IVF study, it significantly increased usable blastocyst rates, highlighting its potential to optimize assisted reproduction.

## Data Availability

The raw data supporting the conclusions of this article will be made available by the authors, without undue reservation.
