# Supplementary material for: Increased reproductive outcomes after optimized sperm preparation
Source: Front Cell Dev Biol. 2025 May 13;13:1596421. doi: 10.3389/fcell.2025.1596421 (PMC12107353; doi:10.3389/fcell.2025.1596421)
Supplement: Supplementary file 1 [file Table1.docx]

**Supplementary Table 1: Kinematic sperm parameters after treatment of mouse sperm with Control or HyperSperm.**

|  | Control | HyperSperm | p-value* |
| --- | --- | --- | --- |
| Motility % | 76.6 ± 20.2 | 77.5 ± 5.6 | 0.625 |
| VCL (μm/s) | 150.3 ± 6.3 | 182.4 ± 23.4 | 0.0489 |
| VSL (μm/s) | 48.7 ± 4.3 | 63.0 ± 12.24 | 0.102 |
| VAP (μm/s) | 80.0 ± 4.0 | 93.6 ± 13.2 | 0.115 |
| LIN (%) | 34.8 ± 3.7 | 35.4 ± 4.7 | 0.736 |
| STR (%) | 61.3 ± 3.0 | 62.7 ± 6.3 | 0.620 |
| ALH (μm) | 10.9 ± 2.3 | 11.6 ± 2.0 | 0.306 |
| BCF (Hz) | 27.7 ± 3.1 | 27.4 ± 4.2 | 0.625 |
| HA (%) | 6.8 ± 2.9 | 16.0 ± 6.4 | 0.0125 |

*Values are expressed as mean ± SD; n=5 independent experiments.*

**Statistical analysis using paired t-test, except for motility and BCF where Wilcoxon signed-rank test was performed. VCL: curvilinear velocity; VSL: straight line velocity; VAP: average path velocity; LIN: linearity; STR: straightness; ALH: amplitude of lateral head displacement; BCF: beat cross frequency; HA: hyperactivation.*
