# Supplementary material for: Increased reproductive outcomes after optimized sperm preparation
Source: Front Cell Dev Biol. 2025 May 13;13:1596421. doi: 10.3389/fcell.2025.1596421 (PMC12107353; doi:10.3389/fcell.2025.1596421)
Supplement: Supplementary file 2 [file Table2.docx]

**Supplementary Table 2. Kinematic sperm parameters after treatment with standard procedures (Control) or HyperSperm of semen samples from normozoospermic donors.**

|  | Control | HyperSperm | p-value* |
| --- | --- | --- | --- |
| Motility (%) | 82.1 ± 9.7 | 89.0 ± 5.8 | 0.0093 |
| Progressive motility (%) | 68.0 ± 17.1 | 83.0 ± 8.7 | 0.0029 |
| VCL (μm/s) | 94.2 ± 17.0 | 132.0 ± 21.2 | 0.0005 |
| VSL (μm/s) | 39.8 ± 14.4 | 51.7 ± 8.5 | 0.0122 |
| VAP (μm/s) | 58.5 ± 13.3 | 73.0 ± 8.5 | 0.0005 |
| LIN (%) | 40.2 ± 10.1 | 40.7 ± 7.4 | >0.999 |
| STR (%) | 60.6 ± 12.8 | 67.0 ± 6.5 | 0.129 |
| WOB (%) | 61.9 ± 5.5 | 57.3 ± 5.5 | 0.0269 |
| ALH (μm) | 2.00 ± 0.28 | 2.75 ± 0.50 | 0.0005 |
| BCF (Hz) | 15.7 ± 2.8 | 18.3 ± 2.1 | 0.0005 |
| HA (%) | 3.02 ± 4.10 | 19.38 ± 13.52 | 0.0005 |

*Values are expressed as mean ± SD; n=12 samples.*

**Statistical analysis using Wilcoxon matched-pairs signed rank test. VCL: curvilinear velocity; VSL: straight line velocity; VAP: average path velocity; LIN: linearity; STR: straightness; WOB: wobble; ALH: amplitude of lateral head displacement; BCF: beat cross frequency; HA: hyperactivation.*
