# Supplementary material for: Increased reproductive outcomes after optimized sperm preparation
Source: Front Cell Dev Biol. 2025 May 13;13:1596421. doi: 10.3389/fcell.2025.1596421 (PMC12107353; doi:10.3389/fcell.2025.1596421)
Supplement: Supplementary file 3 [file Table3.docx]

**Supplementary Table 3. Morphokinetics parameters of embryo development in Control and HyperSperm groups.**

|  | Control | HyperSperm |
| --- | --- | --- |
| t2 (h) | 27.6 (25.2 - 33.0) | 28.1 (25.8 - 31.4) |
| t3 (h) | 39.3 (37.2 - 44.9) | 40.5 (37.1 - 43.1) |
| t4 (h) | 40.3 (37.7 - 46.9) | 41.6 (38.1 - 43.9) |
| t5 (h) | 53.7 (50.9 - 57.8) | 55.4 (51.4 - 58.0) |
| t8 (h) | 59.2 (55.3 - 72.4) | 63.3 (57.3 - 66.7) |
| tM (h) | 94.2 (86.4 - 103.0) | 91.5 (86.5 - 99.3) |
| tB (h) | 106.2 (101.7 - 113.1) | 106.0 (98.1 - 113.6) |
| cc2 (h) | 11.9 (10.9 - 13.3) | 11.9 (11.3 - 13.0) |
| cc3 (h) | 14.0 (12.9 - 15.7) | 14.8 (13.5 - 16.3) |
| s2 (h) | 0.4 (0.2 - 1.1) | 0.8 (0.4 - 1.6) |

*Time to 2 (t2), 3 (t3), 4 (t4), 5 (t5), 8 cells (t8), morula (tM) or blastocyst (tB), duration of the second (cc2 = t3-t2) and third cell cycle (cc3 = t5-t3), and synchronization of the second cell division (s2=t4-t3). Values expressed as median (interquartile range) and analyzed with the log-rank test (Mantel-Cox); n(Control)=17-19 embryos, n(HyperSperm)=29-33 embryos,* *p>0.05.*
